# Novel models to predict elevated intracranial pressure during intensive care and long-term neurological outcome after TBI

**DOI:** 10.1186/cc10898

**Published:** 2012-03-20

**Authors:** F Guiza, B Depreitere, I Piper, G Van den Berghe, G Meyfroidt

**Affiliations:** 1 UZ Leuven, Belgium; 2 Southern General Hospital, Glasgow, UK

## Introduction

Elevated intracranial pressure (ICP) episodes are associated with poor outcome and should be prevented. We developed models to predict these episodes 30 minutes in advance, and to predict long-term neurological outcome by using dynamic characteristics of continuous ICP and mean arterial pressure (MAP) monitoring.

## Methods

The Brain-IT [1] dataset has records for 264 patients from 22 neuro-ICUs in 11 European countries. Logistic regression and Gaussian processes (machine learning method) were used. CRASH [2] and IMPACT [3] predictors were used together with dynamic data.

## Results

**Figure 1:**
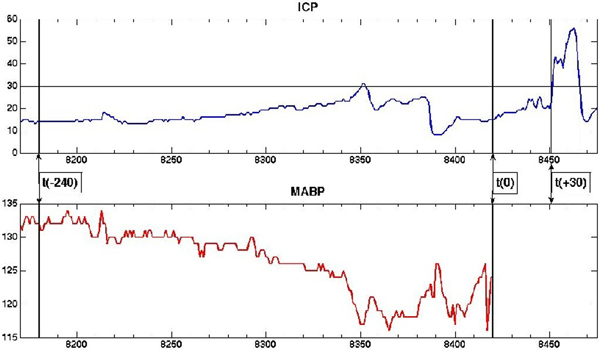
**Elevated ICP episode**.

Predictions of elevated ICP episodes (Figure 1) were externally validated with good calibration and discrimination (AUROC 0.87). Prediction of poor neurological outcome at 6 months (GOS 1 to 2) with static data had 0.72 AUROC; adding dynamic information increased performance to 0.9 (Table 1).

**Table 1:** Model performance

	Elevated ICP	GOS 1 to 2 static	GOS 1 to 2 dynamic
AUROC	0.87	0.72	0.90
HL *P *value	0.12	0.51	0.95
Brier scaled	39.4%	7.7%	46%

## Conclusion

Dynamic data in continuous MAP and ICP monitoring allows prediction of elevated ICP. Adding information of the first 24 hours of ICP and MAP to known risk factors allows accurate prediction of neurological outcome at 6 months.
